# Icariside Ⅱ Attenuates Palmitic Acid-Induced Endothelial Dysfunction Through SRPK1-Akt-eNOS Signaling Pathway

**DOI:** 10.3389/fphar.2022.920601

**Published:** 2022-06-30

**Authors:** Yang-Yang Gu, Xiao-Hui Tan, Wen-Peng Song, Wei-Dong Song, Yi-Ming Yuan, Zhong-Cheng Xin, Jia-Dong Wang, Dong Fang, Rui-Li Guan

**Affiliations:** ^1^ Department of Radiation Medicine, Institute of Systems Biomedicine, School of Basic Medical Sciences, Peking University Health Science Center, Beijing, China; ^2^ Department of Urology, Peking University First Hospital, Beijing, China; ^3^ Institute of Urology, Peking University, Beijing, China; ^4^ Beijing Key Laboratory of Urogenital Diseases (male) Molecular Diagnosis and Treatment Center, Beijing, China; ^5^ Department of Dental Implant Center, Beijing Stomatological Hospital, School of Stomatology, Capital Medical University, Beijing, China; ^6^ Male Reproductive and Sexual Medicine, Department of Urology, The Second Hospital of Tianjin Medical University, Tianjin, China; ^7^ Institute of Urology, Tianjin Medical University, Tianjin, China

**Keywords:** endothelial dysfunction, icariside Ⅱ, palmitic acid, SRPK1-Akt-eNOS signaling pathway, nitric oxide

## Abstract

**Background:** Endothelial dysfunction is commonly accompanied by a reduced capacity for nitric oxide (NO) production and decreased NO sensitivity, playing a central role in numerous vascular diseases. Saturated free fatty acids are known to reduce NO production and then induce endothelial dysfunction. Alternative splicing participates in the regulation of cellular and tissular homeostasis and is highly regulated by serine-arginine protein kinase (SRPK1). The role of SRPK1 in the biology of endothelial cells remains elusive. Icariside Ⅱ (ICA Ⅱ) has been reported to have protective effects on endothelial function. However, the specific molecular mechanisms are still unknown. The purpose of this study is to explore the role of SRPK1 in the biology of endothelial cells and the underlying mechanism of ICA Ⅱ on palmitic acid (PA) induced endothelial dysfunction.

**Methods:** Endothelial dysfunction was induced using PA in human umbilical vein endothelial cells (HUVECs). The expression and phosphorylation of related proteins in the SRPK1-Akt-eNOS signaling pathway were detected by Western Blot. Cell Counting Kit-8 assay and Ki-67 immunofluorescence were used to estimate cell viability. Endothelial cell function was assessed by detecting NO production using DAF-FM DA. Interaction between ICA Ⅱ and SRPK1 was demonstrated by a biotinylated protein interaction pull-down assay.

**Results:** The expressions of eNOS, Akt, and SRPK1 were down-regulated in the endothelial dysfunction stimulated by PA. SRPK1 inhibitor SPHINX31 restrained endothelial cell viability in a dose-dependent manner. Moreover, inhibition of SRPK1 using SPHINX31 and knockdown of SRPK1 by shRNA also showed a down-regulation of the proteins associated with the SRPK1-Akt-eNOS signaling pathway. Biotinylated protein interaction pull-down assay revealed that ICA Ⅱ could be directly bound with SRPK1. On the other hand, ICA Ⅱ could attenuate the PA-induced endothelial dysfunction and restore cell viability through the SRPK1-Akt-eNOS pathway.

**Conclusions:** ICA Ⅱ, bound with SRPK1, could attenuate the endothelial dysfunction induced by the PA in HUVECs *via* the SRPK1-Akt-eNOS signaling pathway.

## 1 Introduction

Endothelial cells (ECs) are a major constitutive part of the heart and vasculature, which form the interface between the vascular lumen and the smooth muscle cells. The well-recognized roles of ECs are to safeguard transport logistics, maintain vascular permeability, mediate immune responses, and regulate vascular tone (Krüger-Genge et al., 2019). Several molecular mechanisms may govern these processes, and the most well-studied one is the nitric oxide (NO) signaling pathway (Incalza et al., 2018). NO is a small, highly reactive, readily diffusible gaseous free radical with strong vasodilatory, anti-inflammatory, and antioxidant properties, playing a central role in the maintenance of vascular homeostasis (Tousoulis et al., 2012). Endothelial dysfunction is commonly accompanied by a reduced capacity for NO production and decreased NO sensitivity, which ultimately leads to an imbalance in vascular homeostasis (Cyr et al., 2020). In endothelial cells, NO is mainly generated by eNOS (Förstermann and Sessa, 2012). Broadly, eNOS catalyze the production of NO and l-citrulline from l-arginine and O_2_, using electrons donated from NADPH (Crane et al., 1998). The expression of eNOS has been critical for the maintenance of appropriate vascular homeostasis, whose activation is mainly regulated by multiple post-translational modifications. The phosphorylation of the Ser1177 residue in the eNOS reductase domain can lead to a higher flux of electrons and promotes the production of NO (Fleming and Busse, 2003). This modification is the end result of multiple signaling cascades. The upstream signaling molecules include protein kinase B (Akt), AMP-activated protein kinase (AMPK), Ca2+/calmodulin-dependent protein kinase II (CaMKII) and protein kinase A. Of note, the principle upstream signaling molecules is Akt (Kolluru et al., 2010). Consequently, eNOS-derived NO signaling pathway is essential for the maintenance of endothelial homeostasis.

Saturated free fatty acids are known to impair eNOS activation, reduce NO production, and then induce endothelial dysfunction (Kim et al., 2005). Additionally, excessive free fatty acids stimulate the generation of free radicals, which impact the functions of important biomacromolecules as well as contribute to cell damage and homeostasis imbalance (Lobo et al., 2010). Palmitic acid (PA) represents the main saturated free fatty acid in the bloodstream and has been used to induce endothelial dysfunction in HUVECs (Lee et al., 2014; Batumalaie et al., 2016). Therefore, PA is a suitable material to establish a model for endothelial dysfunction.

Serine-arginine protein kinase (SRPK1), an enzyme that phosphorylates splicing factors (SR proteins), has a central role in the regulation of alternative splicing (Das and Krainer, 2014). Alternative splicing is the process that removes introns and adds exons in various combinations, resulting in multiple mRNA products and ultimately giving rise to proteins with distinct or even opposing functions (Sciarrillo et al., 2020). Up to now, SRPK1 has been extensively studied in tumors, having a prognostic and potential predictive role in various cancers (Nikas et al., 2019). It has been found that SRPK1 was a genetic vulnerability of acute myeloid leukemia (AML) through effects on isoform usage of epigenetic regulators including BRD4 (Tzelepis et al., 2018). In addition to modulating splicing, SRPK1 could also integrate growth factor signaling in the Akt pathway. Wang et al. noted that both decreased and increased SRPK1 levels promoted cancer by interfering with PHLPP-mediated dephosphorylation of Akt (Wang et al., 2014). Besides, decreased SRPK1 expression led to a decrease in the mRNA level of Akt in vascular smooth muscle cells (Li and Wang, 2019). In contrast, only a few studies have investigated the role of SRPK1 in normal primary ECs. Jia et al. recently demonstrated that FGF-2 promoted angiogenesis by the SRPK1 network that regulated VEGFR1 alternative splicing in ECs (Jia et al., 2021). However, whether SRPK1 is involved in regulating endothelial function remains unclear.

ICA Ⅱ, an active flavonoid, is the main pharmacological metabolite of Icariin that is extracted from the traditional Chinese medicinal *Herba Epimedii* (Xu et al., 2021). ICA Ⅱ possesses multiple biological and pharmacological activities, including anti-inflammatory, anticancer anti-oxidant, anti-aging, and anti-osteoporotic properties (Khan et al., 2015). Additionally, ICA Ⅱ has gradually become an efficient molecule as a natural product for preventing or treating diseases (Chen et al., 2016). Our previous studies demonstrated that ICA Ⅱ could ameliorate endothelial dysfunction by regulating the MAPK pathway and Akt-eNOS signaling pathway in diabetic human cavernous endothelial cells (HCECs) (Li et al., 2015; Lei et al., 2018). Nonetheless, additional research is still required to fully understand the mechanism of therapeutic effects for endothelial dysfunction.

Based on previous research and current knowledge, the purpose of this study is to explore the role of SRPK1 in regulating endothelial homeostasis and the underlying mechanism of ICA Ⅱ on PA-induced endothelial dysfunction in HUVECs.

## 2 Results

### 2.1 SRPK1 Is Down-Regulated in the Endothelial Dysfunction Induced by Palmitic Acid

**FIGURE 1:**
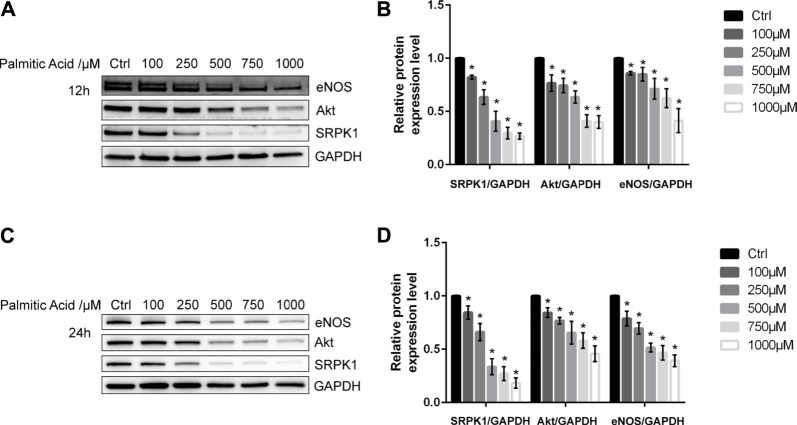
SRPK1 is down-regulated in endothelial dysfunction induced by PA. HUVECs were treated with different concentrations of PA (100, 250, 500, 750, and 1000 μM) for 12 and 24 h. The levels of the eNOS, Akt and SRPK1 were determined by western blot **(A, C)** with the quantifications of the related proteins **(B, D)**. The data are presented as the mean ± SD of three independent experiments. Significance was determined by one-way ANOVA with a least significant difference post hoc test. ns, *p* > 0.05; *, *p* < 0.05 vs Ctrl.

The endothelial dysfunction model was first established in HUVECs. Initially, various concentrations of PA (100, 250, 500, 750, and 1000 μM) were added to HUVECs. Following short-term treatment (12 h) or long-term treatment (24 h), the expression level of eNOS was decreased in a dose-dependent manner, which indicated that the endothelial dysfunction model was successfully established (Figure 1). Besides, we found that SRPK1 and Akt also had similar patterns of expression levels. These results suggested that SRPK1 might be involved in regulating endothelial function.

### 2.2 Effects of SRPK1 on Cell Viability in Human Umbilical Vein Endothelial Cells

**FIGURE 2:**
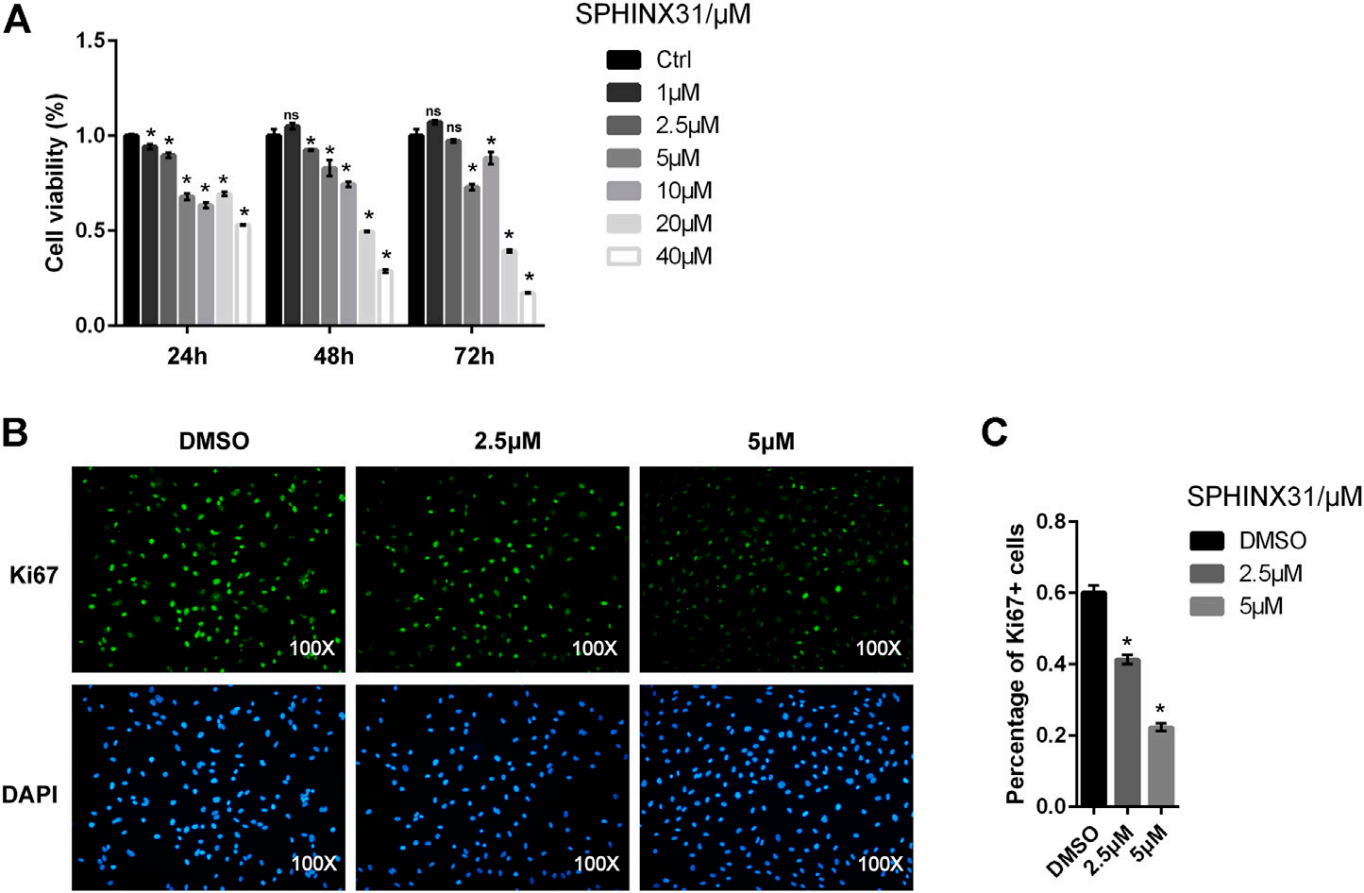
Effects of the inhibitor of SRPK1 on cell viability in HUVECs. HUVECs were treated with different concentrations of SPHINX31 for the indicated time with cell viability measured via CCK-8 **(A)**. After treatment with different concentrations (2.5 and 5 μM) of SPHINX31 for 24 h, the immunofluorescence staining for Ki-67 (green) and DAPI (blue) was determined **(B)**. Images were observed using 100 × fluorescence microscopy with the quantifications of Percentage of Ki-67 + cells **(C)**. The data are presented as the mean ± SD of three independent experiments. Two-way ANOVA with Bonferroni test **(A)** and one-way ANOVA with a least significant difference post hoc test **(C)** were used. ns, *p* > 0.05; *, *p* < 0.05 vs Ctrl.

We first assessed whether SRPK1 affected endothelial cell proliferation or not. The cell viability of HUVECs treated with SPHINX31, a specific inhibitor of SRPK1, was significantly reduced compared with negative controls and exhibited in a dose-dependent manner (Figure 2A). Ki-67 is a nuclear protein expressed in proliferating mammalian cells, regarded as a standard marker of proliferation to assess the growth fraction of a cell population. Treatment with the inhibitor of SRPK1 in the HUVECs led to a significant reduction of the percentage and fluorescence intensity of Ki-67^+^ HUVECs (Figures 2B,C). Overall, inhibition of SRPK1 restrained the proliferation of HUVECs.

### 2.3 Effects of SRPK1 on Endothelial Function in Human Umbilical Vein Endothelial Cells

**FIGURE 3:**
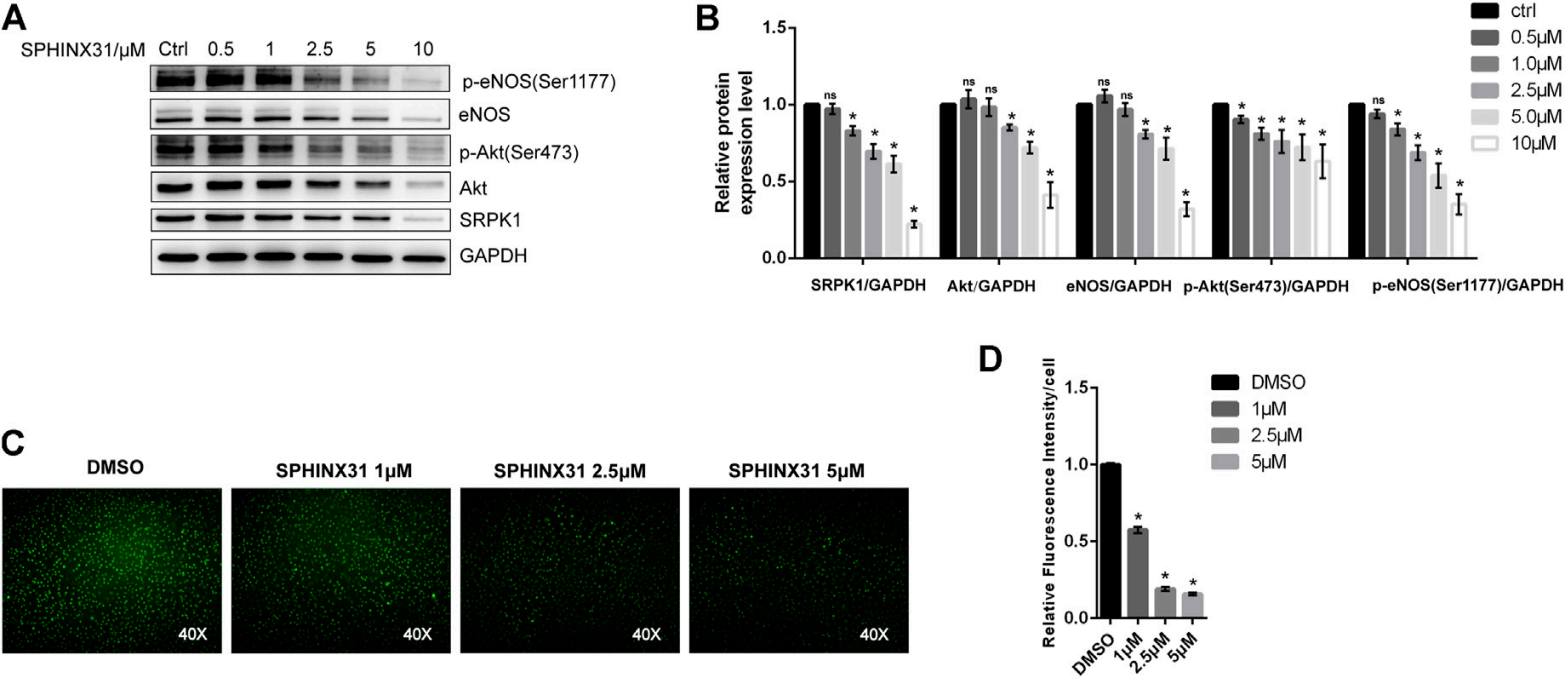
Effects of the inhibitor of SRPK1 on eNOS activity in HUVECs. HUVECs were treated with different concentrations of SPHINX31 for 24 h. The levels of eNOS, phosphor-eNOS (Ser1177), Akt, phosphor-Akt (Ser473), and SRPK1 were determined by western blot **(A)** with the quantifications of the related proteins **(B)**. After treatment with different concentrations (1, 2.5, and 5 μM) of SPHINX31 for 24 h, the immunofluorescence images of the NO probe were observed using 40 × fluorescence microscopy **(C)** with the quantifications of mean fluorescence intensity **(D)**. The data are presented as the mean ± SD of three independent experiments. Significance was determined by one-way ANOVA with a least significant difference post hoc test. ns, *p* > 0.05; *, *p* < 0.05 vs Ctrl.

Next, we investigated the role of SRPK1 in regulating endothelial function with an emphasis on the Akt-eNOS signaling pathway. Interestingly, inhibition of SRPK1 remarkably reduced the expression and phosphorylation of Akt and eNOS (Figures 3A,B), implying that the Akt-eNOS signaling pathway was interdicted by the inhibition of SRPK1. These findings suggested that SRPK1 might act as an upstream signaling molecule in this pathway in ECs. A significant decrease in the production of NO was observed in the SPHINX31-treated HUVECs compared to the control cells (Figures 3C,D).

**FIGURE 4:**
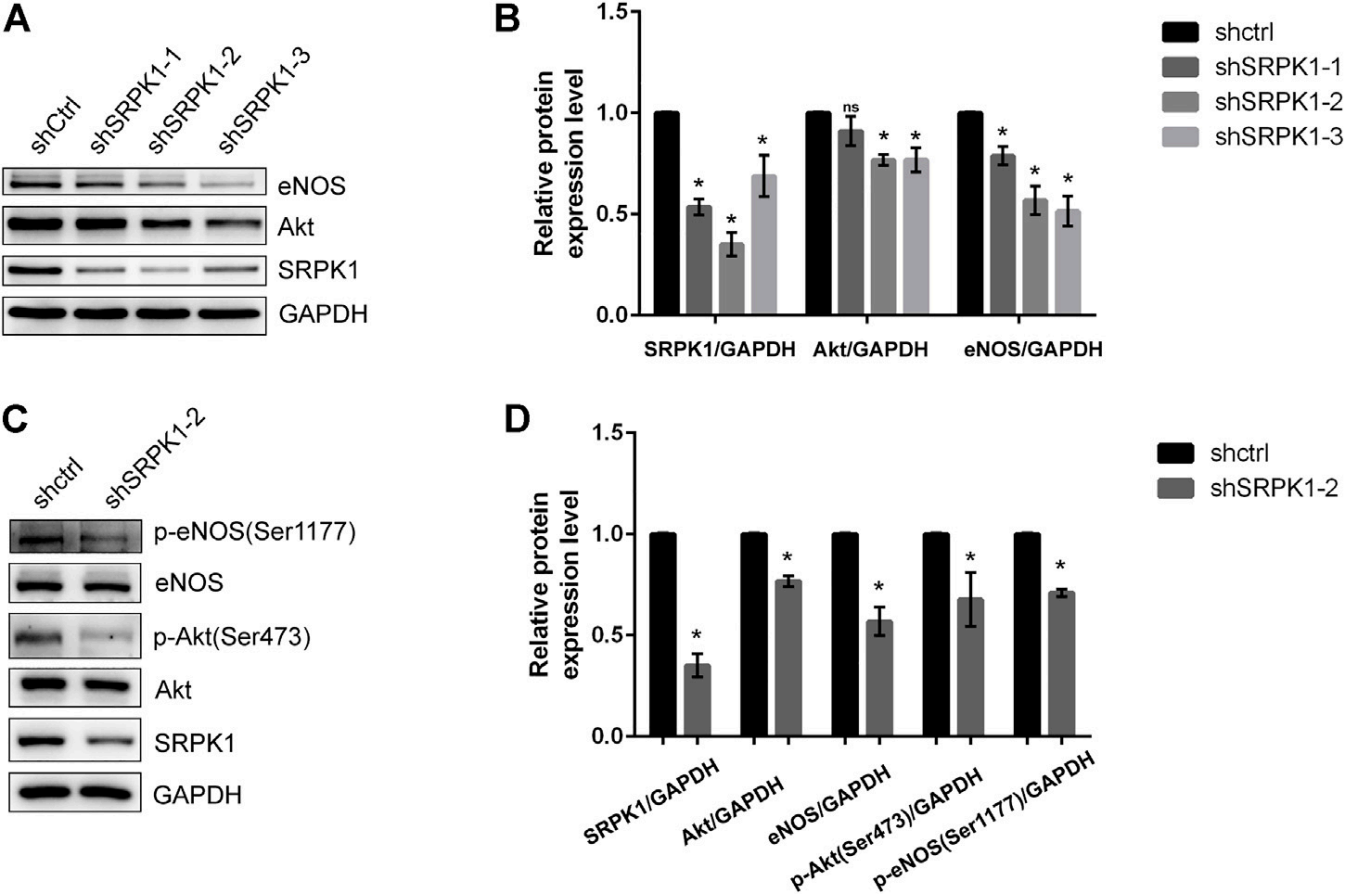
SRPK1 knockdown decreases the expression and phosphorylation of eNOS. HUVECs were transfected with lentiviral shRNA specific for 3 independent sequences of SRPK1 (shSRPK1-1, shSRPK1-2, and shSRPK1-3) and a scrambled shRNA (shCtrl). The levels of the eNOS, Akt and SRPK1 were determined by western blot **(A)** with the quantifications of the related proteins **(B)**. Followed by the transfection with lentiviral shSRPK1-2, the levels of the eNOS, phosphor-eNOS (Ser1177), Akt, phosphor-Akt (Ser473), and SRPK1 were determined by western blot **(C)** with the quantifications of the related proteins **(D)**. The data are presented as the mean ± SD of three independent experiments. One-way ANOVA with a least significant difference post hoc test for **B** and Student’s t-test for **D** were used. ns, *p* > 0.05; *, *p* < 0.05 vs Ctrl.

To further clarify the regulation of SRPK1 on Akt and eNOS, we designed three shRNA sequences with green fluorescence targeting SRPK1 to construct lentiviral vectors for the knockdown of SRPK1 in HUVECs. The second sequence (shSRPK1-2) showed relatively obvious depletion of SRPK1 protein levels (Figures 4A,B). Moreover, SRPK1 knockdown affected the expression levels of Akt and eNOS compared with controls. We also confirmed that the phosphorylation levels of eNOS and Akt were also significantly down-regulated when SRPK1 was knocked down (Figures 4C,D). These findings supported that SRPK1 was necessary to maintain endothelial function via Akt-eNOS signaling pathway.

### 2.4 SRPK1 Is a Direct Icariside Ⅱ-Binding Protein

**FIGURE 5:**
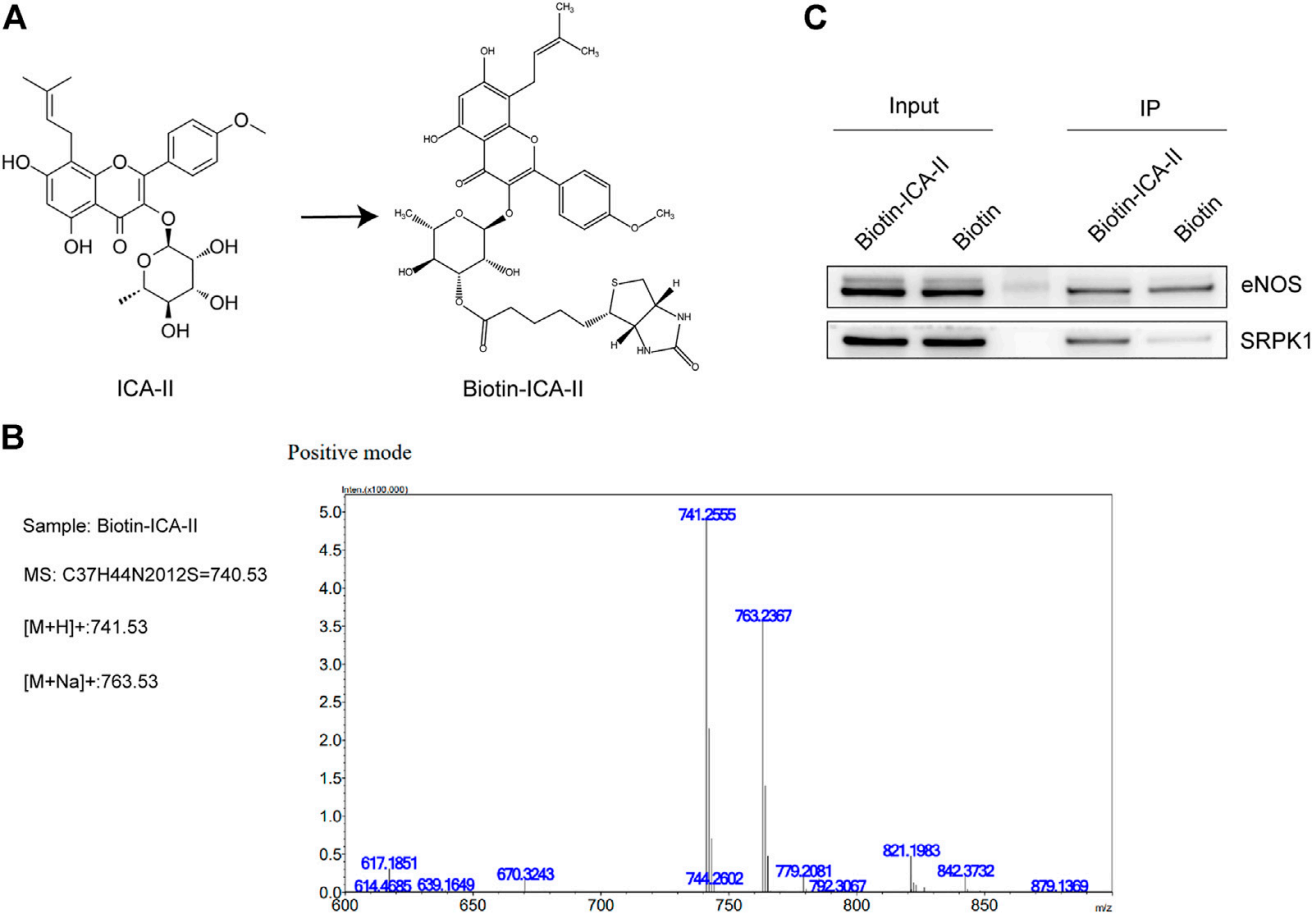
ICA Ⅱ binds SRPK1 protein. **(A)** Chemical name and structure of ICA Ⅱ and Bio-ICA Ⅱ. **(B)** ESI IT-TOF MS fingerprint of Bio-ICA Ⅱ. **(C)** Lysates prepared from HUVECs were examined for ICA Ⅱ binding to SRPK1 and eNOS using pull-down assay, with biotin used as a control.

To clarify whether SRPK1 is involved in the endothelial protective effects of ICA Ⅱ, we studied the binding of ICA Ⅱ and SRPK1 in HUVECs using biotinylated protein interaction pull-down assays. First, ICA Ⅱ was labeled with biotin by means of hydroxyl carboxylation. The chemical structure of ICA Ⅱ and Bio-ICA Ⅱ was shown in Figure 5A and the quality assessment of Bio-ICA Ⅱ was performed using the fingerprinting approach of electrospray ionization time-of-flight mass spectrometry (Figure 5B), suggesting that ICA Ⅱ was successfully labeled with biotin. As a negative control, eNOS had no interaction with Bio-ICA Ⅱ. In contrast, Bio-ICA Ⅱ was bound directly with SRPK1 protein in lysates from HUVECs, showing that SRPK1 could be a target of ICA Ⅱ (Figure 5C).

### 2.5 Icariside Ⅱ Attenuates Palmitic Acid-Induced Endothelial Dysfunction by SRPK1-Akt-eNOS Pathway

**FIGURE 6:**
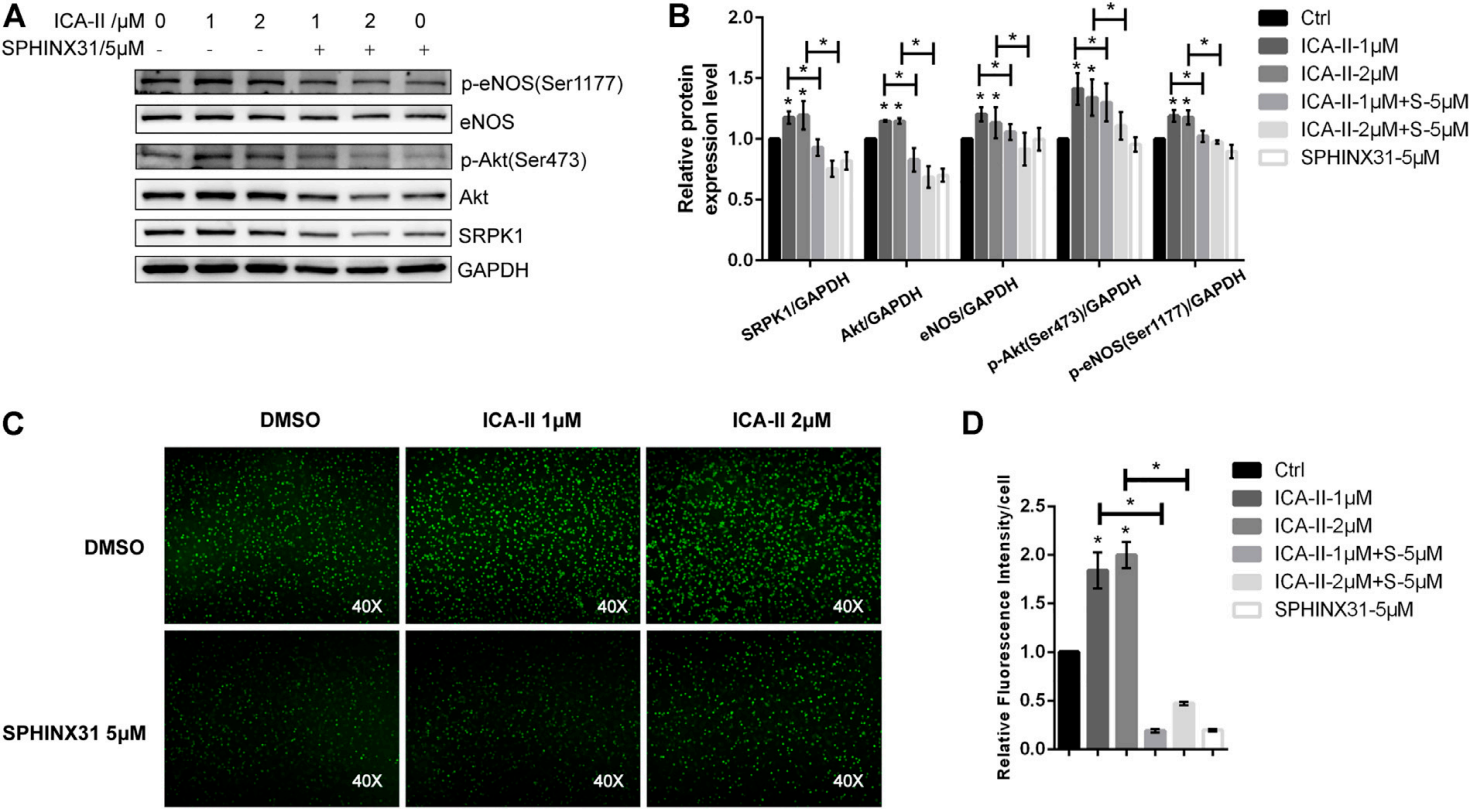
Activation of SRPK1-Akt-eNOS pathway in HUVECs treated with ICA Ⅱ. HUVECs were treated with different concentrations of ICA Ⅱ (1 and 2 μM) in the presence or absence of SPHINX31 (5 μM) for 24 h. The levels of eNOS, phosphor-eNOS (Ser1177), Akt, phosphor-Akt (Ser473), and SRPK1 were determined by western blot **(A)** with the quantifications of the related proteins **(B)**. After the same treatment, the immunofluorescence images of the NO probe were observed using 40× fluorescence microscopy **(C)** with the quantifications of mean fluorescence intensity **(D)**. The data are presented as the mean ± SD of three independent experiments. Significance was determined by one-way ANOVA with a least significant difference post hoc test. ns, *p* > 0.05; *, *p* < 0.05 vs Ctrl.

The binding of Bio-ICA Ⅱ and SRPK1 prompted us to examine whether ICA Ⅱ alleviated PA-induced endothelial dysfunction by targeting SRPK1. We first examined the effects of ICA Ⅱ on endothelial cell function under physiological conditions. Our studies showed that the expressions of SRPK1, Akt and eNOS were slightly up-regulated after treating HUVECs with ICA Ⅱ (1 and 2 μM). Meanwhile, phosphorylation of eNOS at Ser1177 and phosphorylation of Akt at Ser473 were significantly up-regulated, which indicated the activation of SRPK1-Akt-eNOS signaling pathway (Figures 6A,B). Additionally, the NO content in HUVECs treated by ICA Ⅱ was much higher than that in the control group (Figures 6C,D). On the other hand, the expression level and phosphorylation level of related proteins in the SRPK1-Akt-eNOS signaling pathway were blocked by SPHINX31. Consistently, the increased NO induced by treatment with ICA Ⅱ was significantly inhibited by SPHINX31 (Figures 6A–D).

**FIGURE 7:**
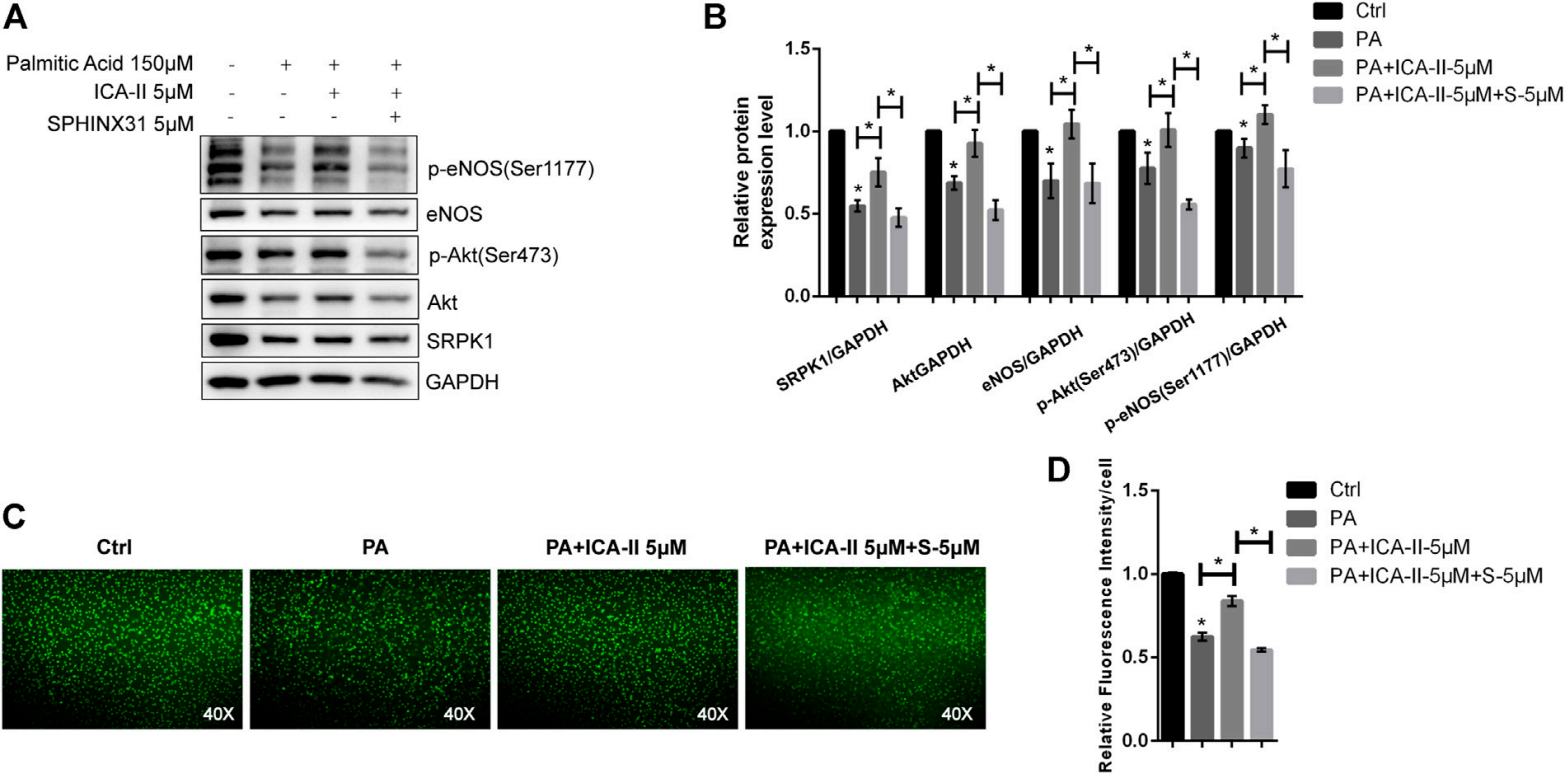
ICA Ⅱ restores SRPK1/Akt/eNOS pathway in PA-treated HUVECs. HUVECs were treated with 5 μM SPHINX31, followed by stimulation with PA (150 μM) in the presence or absence of ICA Ⅱ (5 μM) for 24 h that was pretreated for 1 h. The levels of eNOS, phosphor-eNOS (Ser1177), Akt, phosphor-Akt (Ser473), and SRPK1 were determined by western blot **(A)** with the quantifications of the related proteins **(B)**. The immunofluorescence images of the NO probe were observed using 40 × fluorescence microscopy **(C)** with the quantifications of mean fluorescence intensity **(D)**. The data are presented as the mean ± SD of three independent experiments. Significance was determined by one-way ANOVA with a least significant difference post hoc test. ns, *p* > 0.05; *, *p* < 0.05 vs Ctrl.

Next, we evaluated the effects of ICA Ⅱ on PA-induced endothelial dysfunction. We found that the expression level and phosphorylation level of the proteins associated with SRPK1-Akt-eNOS signaling pathway were significantly down-regulated in the presence of PA but greatly reversed using ICA Ⅱ, indicating a promising therapeutic role of ICA Ⅱ against endothelial dysfunction (Figures 7A,B). More importantly, these suppressed expressions induced by PA were significantly elevated by treatment with ICA Ⅱ but reversed by the inhibition of SRPK1. Furthermore, the generation of NO inhibited by PA was also significantly elevated by treatment with ICA Ⅱ but reversed by the inhibition of SRPK1 (Figures 7C,D). Taken together, these results demonstrated that ICA Ⅱ could attenuate PA-induced endothelial dysfunction via SRPK1-Akt-eNOS pathway.

### 2.6 Icariside Ⅱ Restores Cell Survival Through SRPK1-Akt-eNOS Pathway in Palmitic Acid-Treated Human Umbilical Vein Endothelial Cells

**FIGURE 8:**
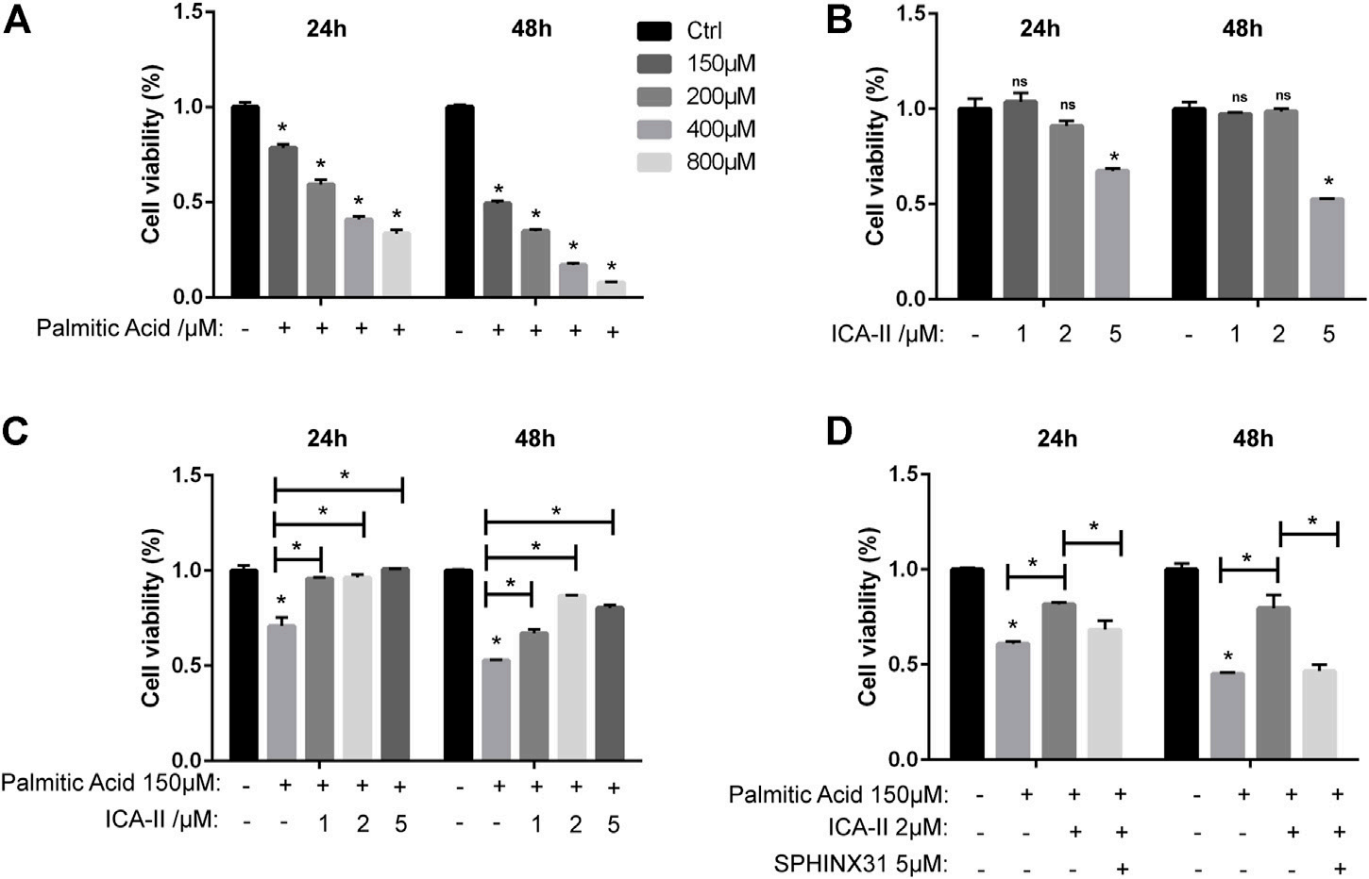
ICA Ⅱ restores cell survival through SRPK1-Akt-eNOS pathway in PA-treated HUVECs. **(A)** HUVECs were treated with different concentrations of PA (150, 200, 400, and 800 μM) for 24 and 48 h with cell viability measured via the CCK-8 assay. **(B)** HUVECs were treated with different concentrations of ICA Ⅱ (1, 2, and 5 μM) for 24 and 48 h with drug toxicity of ICA Ⅱ measured via the CCK-8 assay. **(C)** HUVECs were treated with 150 μM PA in the presence or absence of ICA Ⅱ (1, 2, and 5 μM) for 24 and 48 h with cell viability measured via the CCK-8 assay. **(D)** Cells were treated with 5 μM SPHINX31 and 150 μM PA, followed by the presence or absence of 2 μM ICA Ⅱ for 24 and 48 h with cell viability measured via the CCK-8 assay. The data are presented as the mean ± SD of three independent experiments. Significance was determined by two-way ANOVA with Bonferroni test. ns, *p* > 0.05; *, *p* < 0.05 vs Ctrl.

The CCK-8 assay showed that the cell viability of HUVECs treated with PA was significantly decreased than untreated cells, depending on concentrations (Figure 8A). There was no significant difference between 1 μM or 2 μM ICA Ⅱ and untreated cells (Figure 8B). As expected, the decreased cell viability induced by PA was significantly elevated by treatment with ICA Ⅱ (Figure 8C) but reversed by the inhibition of SRPK1 (Figure 8D). Collectively, these results showed that ICA Ⅱ restored cell survival through SRPK1-Akt-eNOS pathway in PA-treated HUVECs.

## 3 Discussion

**FIGURE 9:**
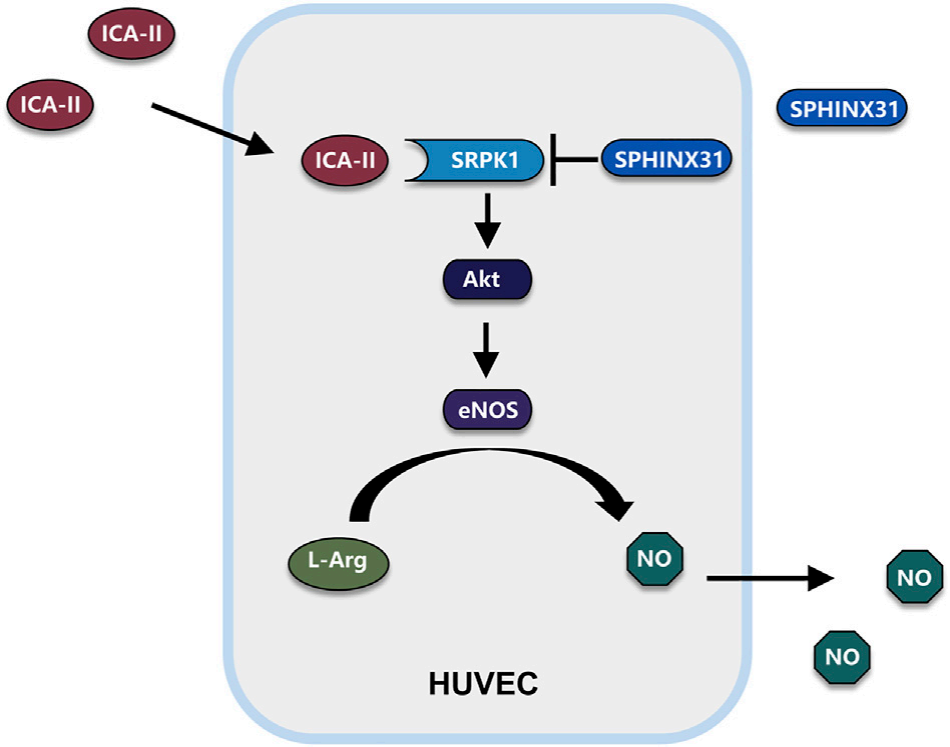
Schematic illustration of potential signaling pathways affected by ICA Ⅱ. Possible mechanism of ICA Ⅱ in the PA-induced endothelial dysfunction by activating SRPK1-Akt-eNOS signaling pathway and promoting the production of NO.

Endothelial dysfunction is commonly accompanied by a reduced capacity for NO production and decreased NO sensitivity, which ultimately results in an imbalance in vascular homeostasis (Cyr et al., 2020). Increasing evidence has demonstrated that endothelial dysfunction is the hallmark of a wide range of vascular diseases associated with vasoconstriction, thrombosis, and inflammatory state (Godo and Shimokawa, 2017). Elevated levels of free fatty acid are considered as an important factor in the onset of endothelial dysfunction. Plasma levels of free fatty acids are increased in subjects with obesity and type 2 diabetes, leading to insulin resistance, inflammation, hypertension, atherosclerosis, and cardiovascular diseases (Boden, 2008; Ghosh et al., 2017). The ability to generate NO has served as a marker for healthy endothelia. Endothelial-derived NO is produced by eNOS and regulates vascular tone. It has been shown that nanomolar concentrations of NO have anti-inflammatory and antioxidant properties (Cyr et al., 2020). In the present study, we demonstrated that ICA Ⅱ protected against PA-induced endothelial dysfunction by activating SRPK1 via the direct interaction between ICA Ⅱ and SRPK1. Meanwhile, PA suppressed the expression of SRPK1, Akt and eNOS, and also inhibited phosphorylation of eNOS at Ser1177 and phosphorylation of Akt at Ser473, followed by the decreased production of NO, which indicated that PA down-regulated the SRPK1-Akt-eNOS signaling pathway. More importantly, ICA Ⅱ ameliorated these harmful effects through the SRPK1-Akt-eNOS signaling pathway (Figure 9).

In our study, SPHINX31, an ATP-competitive inhibitor, could reduce the expression level of SRPK1 similar to shRNA targeting SRPK1. Polier et al. reported a novel mechanism by which ATP competitive inhibitors inhibited kinase activity. They found that ATP-competitive kinase inhibitors could prevent the recruitment of protein kinases to the protein folding system and then lead to the degradation of protein kinases (Polier et al., 2013). We found that either inhibition of SRPK1 with SPHINX31 or knockdown of SRPK1 by shRNA could lead to the down-regulation of Akt and eNOS. The decrease of phosphorylation of Akt and eNOS was mainly due to the down-regulation of Akt and eNOS protein levels because the ratio of phosphorylated form of target protein to total protein decreased slightly without statistical difference (Supplementary Figure S1). However, the underlying mechanisms still need to be further illustrated. Transcripts from nearly all human protein-coding genes undergo one or more forms of alternative splicing. SRPK1, an enzyme that phosphorylates splicing factors rich in serine-arginine dipeptide motifs (SR proteins), has a central role in the regulation of alternative splicing. Originally considered to be devoted to alternative splicing, SRPK1 is now known to expand its influence to additional aspects of mRNA maturation and certain cellular activities such as chromatin reorganization, regulation of cell cycle and p53, and metabolic signaling (Giannakouros et al., 2011). The expression of SRPK1 could be regulated by epigenetics. It has been found that SRPK1 is directly regulated by miR-155, which can inhibit the expression of SRPK1 (Wang et al., 2021). Histone acetylation/methylation and DNA CpG methylation favor either exon skipping or inclusion, mainly through interfering with RNA Pol II-mediated elongation. WT1 exhibits repression of the kinase SRPK1 *via* its zinc fingers domains to DNA, RNA, and proteins (Mohamed et al., 2014). VEGF, a principal angiogenesis factor, contains an alternative 3′ splice site in the terminal exon. VEGF alternative splicing can result either in pro-angiogenic or antiangiogenic isoforms, regulating angiogenesis in cancer (Bowler and Oltean, 2019). Wagner et al. noted that SRPK1 overexpression induced the expression of pro-angiogenic VEGF isoform and vice versa (Wagner et al., 2019). It has been reported that knockdown of SRPK1 and inhibition of SRPK1 prevented angiogenesis and was associated with tumor growth through regulating the alternative splicing of VEGF (Amin et al., 2011). VEGF and its receptor VEGFR2 mediated-signal transduction pathways involve several molecules, including extracellular signal-regulated kinases, Src, phosphoinositide 3 kinase/Akt, focal adhesion kinase, Rho family GTPases, endothelial NO and p38 mitogen-activated protein kinase (Claesson-Welsh and Welsh, 2013). Besides, Xu et al. reported that the downregulation of SRPK1 expression led to a decrease in Akt phosphorylation and inhibited the Akt signaling pathway (Xu et al., 2017) Moreover, a decrease in SRPK1 expression led to a decreased mRNA level of Akt in vascular smooth muscle cells (Li and Wang, 2019). It has also been found that activated Akt could induce SRPK autophosphorylation that switches the splicing kinases from Hsp70-to Hsp90-containing complexes, leading to enhanced SRPK nuclear translocation and SR protein phosphorylation (Zhou et al., 2012). These findings demonstrated an interaction between SRPK1 and Akt. Therefore, SRPK1 may directly or indirectly regulate the expression of Akt and eNOS through alternative splicing, which needs to be further confirmed in the future.

Flavonoids have a number of protective functions such as antioxidative, anti-inflammatory, anti-mutagenic and anti-carcinogenic properties, coupled with their capacity to modulate key cellular enzyme functions (Panche et al., 2016). Several studies have indicated that flavonoids improve endothelial dysfunction, including Quercetin (Kleemann et al., 2011), Apigenin, naringenin (Qin et al., 2016), Epigallocatechin-3-Gallate (Liu et al., 2017), Hesperetin (Rizza et al., 2011), Cyanidin (Sorrenti et al., 2007) as well as ICA Ⅱ. ICA Ⅱ, an active flavonoid, is a natural product from the traditional Chinese medicinal *Herba Epimedii* (Xu et al., 2021)*.* In our previous study, ICA Ⅱ has protective effects on endothelial function in HCECs (Li et al., 2015). In addition to Akt, AMP-activated protein kinase, Ca2+/calmodulin-dependent protein kinase II, and protein kinase A could also be involved in phosphorylation of the Ser1177 residue in the eNOS reductase domain (Kolluru et al., 2010). This modification is the end result of multiple signaling cascades. ICA Ⅱ could ameliorate endothelial dysfunction by regulating the MAPK pathway via miR-126/SPRED1 in HCECs exposed to a diabetic-like environment (Lei et al., 2018). The downstream target of ICA Ⅱ remains unclear at present. Recently, Fu et al. reported that Icariin, the precursor of ICA Ⅱ, was a receptor-binding lead of EGFR (Fu et al., 2021). Nonetheless, the mechanisms of ICA Ⅱ for improving endothelial function still need further investigation. We found that the protective effect of ICA II against endothelial dysfunction induced by PA was significantly eliminated by inhibiting of SRPK1. Furthermore, the interaction between ICA Ⅱ and SRPK1 was confirmed using biotinylated protein interaction pull-down assays, suggesting that SRPK1 was a downstream target of ICA II. It is important to note that SRPK1 may not be the only target of ICA II, as ICA II appears to interact with multiple target proteins. In support of this idea, several studies have shown that ICA Ⅱ regulates many biological functions and signaling pathways, including anti-inflammatory, anticancer anti-oxidant, anti-aging, and anti-osteoporotic properties (Xu et al., 2021). Our study could provide a novel insight into the therapeutic role of ICA Ⅱ. However, several obstacles restrict its further clinical translation, including poor aqueous solubility, low membrane permeability, and obvious efflux from cells. Bovine milk-derived extracellular vesicles are recognized as promising nanoscale delivery to resolve this problem (Tan et al., 2021). In conclusion, we demonstrated that ICA Ⅱ has protective effects and can rescue HUVECs from injury and dysfunction caused by PA *in vitro* via the SRPK1-Akt-eNOS signaling pathway. ICA Ⅱ could be a potential drug for diseases associated with endothelial dysfunction. Further studies are needed to explore the spectrum of flavonoids such as ICA Ⅱ for human health.

## 4 Materials and Methods

### 4.1 Cell Culture and Reagents

Human umbilical vein endothelial cells (HUVECs; #8000, ScienCell, Carlsbad, CA, United States) were cultured in endothelial cell medium (ECM; #1001, ScienCell) containing fetal bovine serum (5%; #0025, ScienCell), endothelial cell growth supplement (1%; #1052, ScienCell), and antibiotic solution (1%, penicillin/streptomycin; #0503, ScienCell). Cells were incubated in a humidified incubator (37°C, 5% CO_2_). Cells at passages 4–8 were used for all experiments. SPHINX31 (#HY-117661, MCE, Shanghai, China), an inhibitor of SRPK1, was dissolved in DMSO and so was Icariside Ⅱ (#HY-N0011, MCE). The solution was stored at -80°C up to 6 months.

### 4.2 Preparation of Palmitic Acid Solution

PA (#HY-N0830, MCE) solution was prepared as described previously (Wang et al., 2020). Briefly, 51.28 mg of PA was completely dissolved in 1.0 ml ethanol to design the stock solution (200 mM). Fatty acid-free bovine serum albumin (#A8850, Solarbio, Beijing, China) solution (10%) was mixed with PA stock solution in the ratio of 1:19 (263 µl: 5 ml) and fully dissolved at 55°C in a water bath. The mixture was filtered with 0.45-µm membranes to prepare PA working solution (10 mM), which was aliquoted into fractions and stored at -80°C.

### 4.3 Western Blot

The HUVECs were lysed in RIPA lysis buffer (#KGP702, Keygen Biotech, Nanjing, China) containing a protease and phosphatase inhibitor cocktail (#PPC1010, Sigma-Aldrich, St. Louis, MO, United States). Protein concentrations were determined using a BCA protein assay kit (Beyotime Biotech, Shanghai, China). Equal amounts of proteins were electrophoresed using sodium dodecyl sulfate-polyacrylamide gel and then transferred to a polyvinylidene fluoride membrane (Millipore, Bedford, MA, United States). After being blocked with 5% skimmed milk for 1 h at room temperature, the membranes were incubated overnight at 4°C with primary antibodies against eNOS (1:1000; #A1548, ABclonal Technology, Woburn, MA, United States), Akt (1:1000; #2920S, Cell Signaling Technology, Danvers, MA, United States), SRPK1 (1:2000; #611072, BD Bioscience, San Jose, CA, United States), Phospho-eNOS (Ser1177) (1:1000; #9571S, Cell Signaling Technology), Phospho-Akt (Ser473) (1:1000; #4060S, Cell Signaling Technology) and GAPDH (1:20,000; #60004-1-Ig, Proteintech, Rosemont, IL, United States). The membrane was immersed in an HRP-conjugated secondary antibody, followed by chemiluminescence detection using the Syngene G-Box (Syngene, Cambridge, United Kingdom). The band intensity was quantified using ImageJ software (NIH, Bethesda, MD, United States) and normalized with GAPDH as an internal control. The reliability of antibodies used in this study has been verified by many literatures such as eNOS (Luo et al., 2017), SRPK1 (Zhou et al., 2012), Akt, Phospho-Akt (Ser473), Phospho-eNOS (Ser1177) (Kumari et al., 2021) and GAPDH (Leto et al., 2019).

### 4.4 Immunoprecipitation and Co-immunoprecipitation

To assess the binding of ICA Ⅱ to SRPK1, we utilized a protein-interaction pull-down assay with Pierce™ Biotinylated Protein Interaction Pull-Down kit (#21115, ThermoFisher Scientific, Waltham, MA, United States). Three hundred µL of 5 mM biotinylated-ICA Ⅱ was added to 50 µL streptavidin-agarose beads and incubated at 4°C for 1 h. Biotin alone was used as a control. Then 250 µL of Biotin Blocking Solution was added to each spin column and incubated at room temperature for 5 min. Lysates prepared from HUVECs were then added to the streptavidin-agarose beads with bio-ICA Ⅱ. The mixture was incubated at 4°C for 24 h with gentle rocking. Samples were then spun and washed 3 times. The elution buffer was added to each spin column. The eluent was boiled with 5 x loading buffer, and the samples were loaded on a 10% polyacrylamide gel for Western Blot analysis. Total lysates were used as input control.

### 4.5 Immunofluorescence Staining

HUVECs were seeded in 6-well culture plates and immunofluorescence stained in chamber slides. After exposure to SPHINX31, cells were fixed with 4% cold paraformaldehyde for 15 min, immersed in 0.25% Triton X-100 for 5 min, blocked with 1% BSA for 30 min, and incubated with anti-Ki67 (1:100 in 1% BSA; #27309-1-AP, Proteintech) for 60 min at room temperature or overnight at 4°C. After washing three times with PBS, the cells were then incubated using fluorescein-conjugated secondary antibodies (1:200 in 1% BSA, Proteintech) for 1 h at room temperature. Following the staining with 4′,6-diamidino-2-phenylindole (DAPI) for 5 min to counterstain cell nuclei, the cell images were captured using a digital camera (Leica DM2500, Leica Microsystems, Wetzlar, Germany).

### 4.6 Cell Viability Measurement

The cell viability was measured using the Cell Counting Kit-8 assay (CCK-8; #KGA317, Keygen Biotech). Briefly, HUVECs were seeded in 96-well plates (3  ×  10^3^ cells per well) and cultured for 12 h. The medium was replaced with a complete medium containing different concentrations of drugs and incubated for the indicated time. After incubation with CCK-8 solution at 37°C for 2 h, absorbance at 450 nm was determined with a microplate reader (ThermoFisher Scientific). Effects on cell viability were assessed as the percent cell viability compared with that in the untreated control group, which was arbitrarily considered 100% viability.

### 4.7 Construction of shRNA

Human SRPK1 shRNAs were cloned into the lentiviral vector pHS-ASR (pLV-hU6-hef1a-mNeongreen-P2A-puro; Syngentech, Beijing, China) to generate recombinant plasmids. Designed shRNA sequences were listed as follows:

shSRPK1-1: GAA CAA CAC ATT AGC CAA CTT;

shSRPK1-2: CCA GGC AGA ATT ACT AGA GAA;

shSRPK1-3: CAG ACC CTA ATG ATC CAA ATA; The complementary oligonucleotides were first phosphorylated by T4 polynucleotide kinase (New England Biolabs, Ipswich, MA, United States) in T4 DNA ligase buffer containing ATP (New England Biolabs) for 30 min at 37°C. Subsequently, the annealing reactions for each pair of oligonucleotides were performed at 95°C for 5 min and gradually cooled down to room temperature using a thermal cycler, which enables the reaction to ramp down to 25°C at 5°C/min. At the same time, the backbone vector was digested with Bsal to create the overhangs before gel purification. Then, 1 µL annealed product was used to ligate to at least 50 ng of the digested vector before the transformation was performed in M5 HiPer DH5*α* Competent Cells (Mei5 Biotechnology, Beijing, China).

### 4.8 Packaging of Lentivirus

For lentiviral production, the HEK 293T cell line was co-transfected with the PMD2G, psPAX2 and shSRPK1 plasmids in the ratio of 1:3:4 using jetOPTIMUS^®^ DNA transfection Reagent (#117–15; Polyplus, Illkirch, France). The transfected HEK 293T cells were replaced with antibiotic-free medium after 4–6 h. Lentivirus was collected after continuing to culture for 48 h at 37°C, 5% CO2. The collected virus supernatant was filtered by a 0.45um filter, and stored at -80°C for subsequent use.

### 4.9 Measurement of Nitric Oxide Production

HUVECs were seeded in 96-well plates (2  ×  10^4^ cells per well) and cultured for 12 h. The medium was added with different amounts of drugs and incubated for 24 h. Intracellular content of NO was detected by the probe 3-Amino, 4-aminomethyl-2′,7′-difluorescein, diacetate (DAF-FM DA; Beyotime Biotech, Shanghai, China) according to the manufacturer’s protocol. Intracellular NO was examined in HUVECs loaded with the NO-sensitive fluorescent probe DAF-FM DA (5 μM) at 37°C for 30 min. Samples were monitored under a digital camera (Leica DM2500, Leica Microsystems, Wetzlar, Germany).

### 4.10 Statistical Analysis

Statistical analyses were performed using GraphPad Prism, version 9.0 (GraphPad Software, San Diego, CA, United States). Results were presented as mean ± standard deviation. Differences between two groups were determined using unpaired Student’s t-test. Additionally, one-way ANOVA with a least significant difference post hoc test was used to compare mean values between multiple groups, and a two-tailed, two-way ANOVA was utilized in multiple comparisons, followed by the Bonferroni post hoc analysis to identify interactions. *p*-values were considered statistically significant if less than 0.05.

## Data Availability

The original contributions presented in the study are included in the article/Supplementary Material, further inquiries can be directed to the corresponding authors.
