# Supplementary material for: Icariside Ⅱ Attenuates Palmitic Acid-Induced Endothelial Dysfunction Through SRPK1-Akt-eNOS Signaling Pathway
Source: Front Pharmacol. 2022 Jun 30;13:920601. doi: 10.3389/fphar.2022.920601 (PMC9280058; doi:10.3389/fphar.2022.920601)
Supplement: Supplementary file 1 [file DataSheet1.PDF]

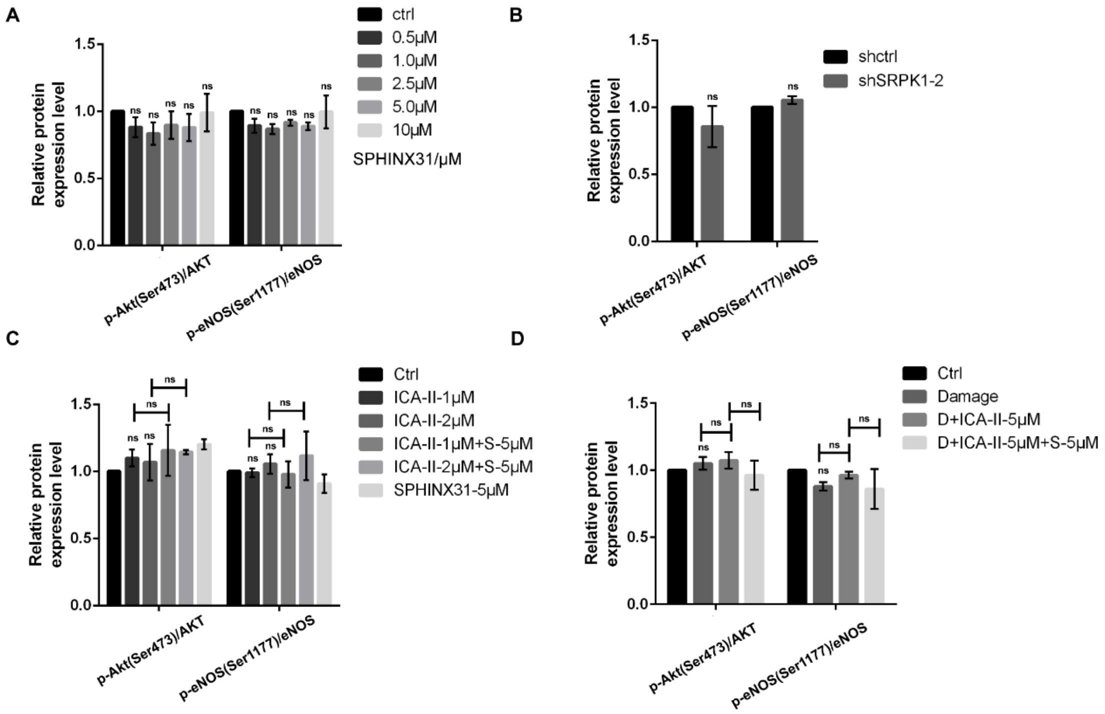


**Figure S1 ratio of phosphorylated form / total protein for eNOS and Akt**

**(A)** Quantifications of figure 3A. **(B)** Quantifications of figure 4C. **(C)** Quantifications of figure 6A. **(D)** Quantifications of figure 7A. The data are presented as the mean ± SD of three independent experiments. One-way ANOVA with a least significant difference post hoc test for A,C,D and Student's t-test for B. ns, $P > 0.05$; *, $P < 0.05$ vs. Ctrl.
